# Evaluation of a site selection questionnaire for the recruitment of trial sites into multi-centre trials: experiences from the nottingham clinical trials unit

**DOI:** 10.1186/1745-6215-14-S1-O30

**Published:** 2013-11-29

**Authors:** Diane Whitham, Lelia Duley

**Affiliations:** 1 University of Nottingham, Nottingham, UK

## Background

Careful site selection methods and tools such as questionnaires have evolved to become "best" practice in the commercial and non-commercial clinical trials setting. However, there is little evidence in the literature of the value of such methodology on trial delivery and there is no generally acceptable model or tool available for use in site selection. The Nottingham Clinical Trials Unit introduced a site selection questionnaire in 2010, consisting of both generic research related questions and study specific protocol driven requirements for identification of potential sites in four multi-centre trials. Initially questionnaires were sent to Principal Investigators at sites who had expressed an interest in participating in the trials.

## Method

The site selection questionnaire was assessed based on the number of questionnaires sent out, return rates, timeliness of returns, completeness of data, and performance against predicted recruitment targets were also compared.

## Results

Across the four multi-centre trials involved there have been 270 questionnaires distributed and 95 sites selected to two of the trials. The total number of sites to be selected is 195. Results will be presented and discussed.

## Discussion

Site selection is a key element underpinning the efficient and successful delivery of clinical trials. Results from this evaluation will contribute to the understanding of how site selection questionnaires might improve trial conduct for trials involving a large number of centres.

